# Assessment of Breathing Parameters Using an Inertial Measurement Unit (IMU)-Based System

**DOI:** 10.3390/s19010088

**Published:** 2018-12-27

**Authors:** Ambra Cesareo, Ylenia Previtali, Emilia Biffi, Andrea Aliverti

**Affiliations:** 1 Dipartimento di Elettronica, Informazione e Bioingegneria, Politecnico di Milano, 20133 Milan, Italy; ambra.cesareo@polimi.it (A.C.); ylenia1.previtali@mail.polimi.it (Y.P.); andrea.aliverti@polimi.it (A.A.); 2 Scientific Institute, IRCCS E. Medea, Bioengineering Lab, 23842 Bosisio Parini, Lecco, Italy

**Keywords:** principal component analysis, biomedical signal processing, wearable biomedical sensors, wireless sensor network, respiratory monitoring, optoelectronic plethysmography

## Abstract

Breathing frequency (f_B_) is an important vital sign that—if appropriately monitored—may help to predict clinical adverse events. Inertial sensors open the door to the development of low-cost, wearable, and easy-to-use breathing-monitoring systems. The present paper proposes a new posture-independent processing algorithm for breath-by-breath extraction of breathing temporal parameters from chest-wall inclination change signals measured using inertial measurement units. An important step of the processing algorithm is dimension reduction (DR) that allows the extraction of a single respiratory signal starting from 4-component quaternion data. Three different DR methods are proposed and compared in terms of accuracy of breathing temporal parameter estimation, in a group of healthy subjects, considering different breathing patterns and different postures; optoelectronic plethysmography was used as reference system. In this study, we found that the method based on PCA-fusion of the four quaternion components provided the best f_B_ estimation performance in terms of mean absolute errors (<2 breaths/min), correlation (r > 0.963) and Bland–Altman Analysis, outperforming the other two methods, based on the selection of a single quaternion component, identified on the basis of spectral analysis; particularly, in supine position, results provided by PCA-based method were even better than those obtained with the ideal quaternion component, determined a posteriori as the one providing the minimum estimation error. The proposed algorithm and system were able to successfully reconstruct the respiration-induced movement, and to accurately determine the respiratory rate in an automatic, position-independent manner.

## 1. Introduction

Continuous monitoring of respiratory parameters such as breathing frequency (f_B_), inspiratory time (T_I_) and expiratory time (T_E_) could foster early diagnosis of a wide range of respiratory disorders and help to track a patient’s condition, discriminating between stable and at-risk patients [1,2]. Conditions of interest could be sleep breathing disorders, sudden infant death syndrome, chronic obstructive pulmonary disease (COPD) and neuromuscular disorders. The current gold standard for measuring f_B_ is to count the number of breaths in one minute, through auscultation or observation [3,4]. Other methods for breathing function assessment currently used in clinical practice are spirometry or pneumotachograph based on airflow measurement by using mouthpiece or facemask. In overnight polysomnography, breathing activity is assessed both by measuring respiratory flow, through pressure transducer or thermistors near the nostrils, and respiratory efforts (breathing-derived chest-wall movements), by strain-gauge belts. Also, exhaled carbon dioxide sensors, transthoracic inductance and impedance plethysmography and ECG—or PPG—derived f_B_ have been used to measure breathing signal. Despite their accuracy, these methods are uncomfortable and intrusive, and are not suitable for continuous monitoring in the clinical environment and at home. An emerging area of interest is to use motion sensors to detect the small breathing-derived movements/orientation changes of the chest wall. This method is particularly suitable for long-term breathing monitoring because it is unobtrusive, tolerable, and low-cost. The principle was first presented with a single-axis accelerometer in animal model (dog) using a pressure transducer in the trachea as reference [5]. Starting from this point, a variety of studies demonstrated the feasibility of using one accelerometer placed on the chest wall to derive respiratory signal and/or breathing frequency in different positions [6,7,8,9,10,11,12,13,14]. Morillo et al. [8] combined a piezoelectric single-axis accelerometer and a polarized capacitive microphone placed on the suprasternal notch to collect information of the cardiac, respiratory, and snoring activities for the screening of patients affected by Sleep Apnea-Hypopnea Syndrome. Measurements were limited to the supine position, that was selected to increase the sensitivity of the single-axis accelerometer, limiting the generality of the findings. The analysis method was based on the estimation of breathing frequency through the identification of the peak of the spectrum or autocorrelation; the main limitation of this approach is that, when the breathing is irregular, a main peak may not exist, and individual breaths must be identified and counted. Hung et al. [7] moved from single-axis to biaxial accelerometers. The aim of their study was to evaluate the reliability of the device in terms of detection of the onsets of expiration and expiration, and to assess the feasibility of differentiating between different breathing patterns (normal breathing, apnea, deep breathing). The signals from both axes (anteroposterior and longitudinal) of the accelerometer were summed, limiting the analysis to the sagittal plane, in sitting and lying positions. An adaptive band-pass filter was applied with a variable passband centered at the detected dominant breathing frequency.

As emerged by these studies, single or dual-axis accelerometers can be used to derive breathing signal when appropriately aligned with the major axis of rotation, which changes when the subject move from a posture to another. Contrarily, the use of a tri-axial accelerometer allows measuring inclination changes due to breathing regardless of orientation. In this case, the problem lies in the identification of the accelerometer axis to consider when posture changes. Bates et al. [13] proposed a method to track the major axis of rotation as it changes, to continuously monitor angular motion due to breathing also when subject change position/orientation. An alternative possibility to the best axis selection is fusing the axes. Jin et al. [12] proposed a posture-independent signal processing method based on three possible algorithms for accelerometer axes fusion. They demonstrated that methods based on Principal Component Analysis (PCA) obtained the highest performance in terms of Signal-to-Noise Ratio (SNR), but no results were provided about breathing rate estimation or validation against a reference method.

With the entry of tri-axial accelerometers new opportunities opened for the monitoring of breathing frequency using inertial sensors, but their use was still confined to static conditions since, when the subject is moving, the degree of the movement-related signal would exceed that due to breathing. One possible approach is to identify non-breathing motion, as proposed by Bates et al [13]. In a successive study, Mann et al. [11] furtherly developed the method proposed by Bates et al. [13], by adding activity tracking, and allowing identification of asymmetric breaths, that was not possible in the original method. An attempt to remove motion artifacts by using signal processing was made by Liu et al. [14]. They proposed an elegant method based on PCA-fusion of the three axes of an accelerometer and on filtering of the first principal component by using an adaptive filter that varied according to the energy expenditure derived by the same accelerometer. To overcome the problems of using a single accelerometer in dynamic conditions, a possibility is to fuse data from accelerometers and from other sensors, such as gyroscopes. Yoon et al. [15] investigated the feasibility of measuring breathing-related motions also during dynamic activities of the subject, by fusing data from a tri-axial accelerometer and a gyroscope and applying Kalman filter. They found that, during dynamic exercises, fusion of accelerometer and gyroscope data provided benefits in terms of reduction of estimation error. Gollee et al. used a more complex system, an inertial measurement unit (IMU) fusing accelerometer, gyroscope and magnetometer but considered only static conditions [16]. Another approach to overcome the problems related to motion artefacts is modularity. Lapi et al. [17] tried to overcome limitations deriving from the use of a single accelerometer by proposing a system based on a couple of 3-axis accelerometers placed bilaterally on the chest. Using two accelerometers permitted to detect respiration-related chest-wall movements regardless of sensor positioning with respect to the gravity vector; secondly, the breathing frequency can be obtained even when one of the two sensors is silenced by postural constraints. Recently, Gaidhani at al. [18] proposed a method that uses two IMUs composed by a 3-axis accelerometer, a 3-axis gyroscope and a 3-axis magnetometer, placed on the anterior and posterior side of the chest to decompose the motions experienced by the two IMUs into trunk movements and breathing actions. This paper presents an automatic processing algorithm to derive breathing frequency and other breathing temporal parameters from quaternion-based orientation signals recorded simultaneously at thoracic and abdominal level by using a modular, wireless, IMU-based device [19]. An important step of the processing algorithm is dimension reduction (DR) that allows the extraction of a single respiratory signal starting from 4-component quaternion data. Three different methods of DR are proposed and compared; two of them are based on the selection of one quaternion component, the third one is based on PCA-fusion of the 4 quaternion components. Results obtained using the IMU-based device, with the three different methods, are validated against optoelectronic plethysmography, an already established method to evaluate ventilation through an external measurement of the chest-wall surface motion [20,21,22,23,24,25].

## 2. Materials and Methods

### 2.1. Device Architecture and Hardware Description

The system used in this study is composed by three IMU-sensor units that communicate via Bluetooth with a smartphone; here data are pre-processed and saved. Two of the three sensor units (peripheral units) are dedicated to the recording of chest-wall respiratory-related movements and are placed on the thorax and on the abdomen to record respiratory information about both the compartments; the third sensor unit (reference central unit) is placed on a body area that is integral with the chest wall, but not involved in respiratory movements (e.g., coccyx or anterior superior iliac crest). The measurement of chest-wall movements, related to both abdominal and thoracic compartments, allows the consideration of the two-degree-of-freedom (DoF) model of chest-wall breathing movements [26], that considers abdomen and rib cage (thorax) as acting independently. Moreover, the compartmental contribution to total chest-wall volume changes according to posture and the breathing strategy adopted by each subject. Thus, the recording of chest-wall movements at different levels provides on the one hand, a more accurate estimation of the breathing signal, and on the other hand allows investigation of asynchronies between compartments, typical of different pathological conditions. The reference central unit, in addition to performing a central role within the Bluetooth piconet, can be used to discriminate between static and dynamic conditions and to map the activity state of the subject. Moreover, it could be used to reduce movement information not linked to breathing by means of frequency domain analysis or by referring orientation change experienced by the peripheral units to the coordinate frame of the reference unit. Each unit is composed by a printed circuit board equipped with a low-power microcontroller, a Bluetooth Low Energy (BLE) module, a 9-DoF IMU (3-axis accelerometer, a 3-axis gyroscope, and a 3-axis magnetometer) and lithium polymer rechargeable battery. A voltage regulator circuit, and Li–Po battery recharge circuit with mini USB port are also included in the design. Differently from the peripheral units, the reference central unit is equipped with a different BLE module, able to support simultaneous central/peripheral role and also brings a Micro Secure Digital (SD) Memory Card Connector for data logging. The dimensions of each peripheral unit, comprehensive of the 3D-printed housing, are 41 mm × 33 mm× 19 mm (LWH), and the weight is 25 g, including the battery, while the reference central unit measures 45 mm × 45 mm × 15 mm (LWH), and weighs 35 g. A prototypal version of this device has been described in [19].

### 2.2. Quaternion-Based Orientation Estimation and Fusion Algorithm 

The final goal is to derive breathing signal by measuring orientation changes during the respiratory movements, both at thoracic and abdominal level. The IMUs provide 3D-acceleration, 3D-magnetic field, and 3D-angular rate. These measures are combined to provide accurate 3D orientation data aboard each unit. The orientation is represented with quaternions [27,28], that even though may suffer from problems of interpretation in terms of meaningfully physical angles, are interesting mathematical entities (four-dimensional complex number (q = [q_0_ q_1_ q_2_ q_3_]), since they require less computing time and avoid the singularity problems (i.e., “gimbal lock”) typical of other orientation descriptors, e.g., Euler angles. The fusion of the data collected from the sensors is done by using the sensor fusion algorithm proposed by Madgwick et al. [29], based on an analytically derived and optimized gradient descent algorithm enabling levels of accuracy exceeding that of the Kalman-based algorithm, with low computational (277 scalar arithmetic operations each filter update) load and low sampling rates (e.g., 10 Hz); this orientation filter also provides an online magnetic distortion compensation algorithm and gyroscope bias drift compensation. The sensors data were collected at 40 Hz and the fusion algorithm was updated with the same rate, but due to limited buffer of the BLE module and to the stricter timings used for the Bluetooth communication, just one quaternion out of 4 computed is considered (10 Hz); nevertheless, the final sampling rate was considered appropriate given the relative low frequency of the respiratory signal [0.1 ÷ 1 Hz]. Thus, the microprocessor of each unit, receives data from accelerometer, gyroscope and magnetometer that are on board and implements Madgwick fusion filter [29] to compute a quaternion representing the change of orientation of each unit relative to the earth frame (q^EarthTh, q^EarthAb, q^EarthRef), or more correctly the change of orientation of the earth relative to each unit frame [29]. In fact, in quaternion form, an arbitrary orientation of a coordinate frame B relative to coordinate frame A, achieved through a rotation of angle θ around an axis ^A^**r** (r_x_, r_y_, r_z_) defined in frame A, is univocally represented through the normalized quaternion q^BA defined by Equation (1):

(1) q^BA=[q0 q1 q2 q3]=[cosθ2−rx sinθ2−ry sinθ2 rz sinθ2]

### 2.3. Quaternion-Derived Breathing Frequency

**Figure 1:**
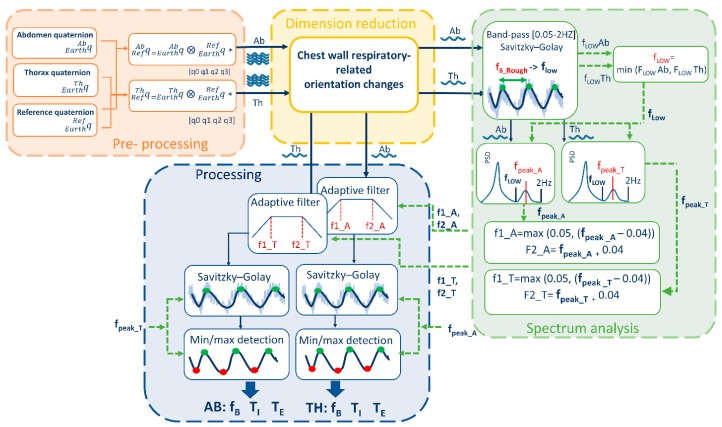
Block Diagram of the Analysis algorithm that allows derivation of breathing temporal parameters (f_B_. T_I_, T_E_) from quaternion-based orientation change signals recorded on Thorax, Abdomen and Reference point.

All the elaborations and computations needed to extract breathing parameters from data collected by the device were performed offline using MATLAB, the processing took on average 1.027 ± 0.129 seconds for the analysis of signals of 1071 ± 270 samples. A signal processing procedure was designed to extract the breathing frequency starting from quaternions representing the change of orientation of each unit relative to the earth frame (q^EarthTh, q^EarthAb, q^EarthRef). The block diagram of the signal processing part is presented in Figure 1. The algorithm is divided into 4 main blocks: (i) pre-processing, (ii) DR, (iii) spectrum analysis, and (iv) processing.

**Pre-processing** block includes the preliminary steps that leads to chest-wall respiratory-related orientation change signals. The orientations changes of thoracic and abdominal units were referred to the reference unit frame (that in turn represents orientation changes of trunk) applying Equations (2) and (3) respectively:

(2) q^RefTh=q^EarthTh ⨂ q^*EarthRef =q^EarthTh ⨂ q^RefEarth,

(3) q^RefAb=q^EarthAb ⨂ q^*EarthRef =q^EarthAb ⨂ q^RefEarth,

These two quaternions represent the outputs of the pre-processing block and the input of the DR block.

**Figure 2:**
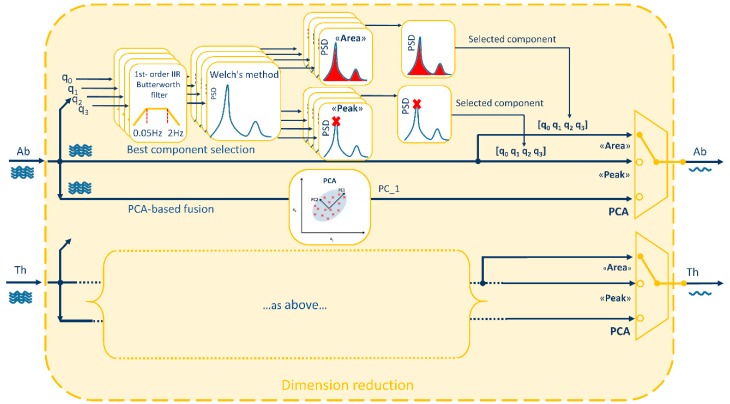
Dimension-reduction block in detail. Starting from the 4 components [q_0_, q_1_, q_2_, q_3_] of each quaternion (Abdominal: Ab and Thoracic: Th), three methods are applied to obtain a single-component signal: two methods based on best quaternion component selection (“Area” and “Peak”) and one method based on the fusion of the 4 components through Principal Component Analysis (PCA). “Area” method selects the quaternion component with the larger area under the Power Spectral Density (PSD) estimate, while “Peak” method selects the quaternion component with the highest PSD’s peak. PCA-fusion method selects the first principal component (PC_1) that accounts for the largest variance in the data.

**Dimension-reduction** block takes the quaternions obtained from Equations (2) and (3), that are composed by 4 components each, and provides as output 2 single-component signals (1 for the abdomen and 1 for the thorax) representing chest-wall respiratory-related orientation change signals. These two signals represent the input of the power spectrum block and of the processing block. To reduce dimension from 4 components to 1, two possibilities were investigated as shown in Figure 2:

(i). Best quaternion component selection
(ii). PCA-based fusion of the quaternion components

To select the best component among the 4 components representing the orientation quaternion, two different methods were proposed, both based on spectrum analysis. The idea was to choose the component with the highest breathing information, computing the power spectral density estimate (PSD) between 0.5–2 Hz for each component and selecting the component with: (1) maximum PSD peak (“Peak” method) or (2) maximum area under the PSD (“Area” method). To assess the goodness of these two methods in predicting the best quaternion component, the ideal component (“Ideal”) was determined a posteriori, case by case, based on minimum breathing frequency estimation error (see Section 2.5).

Since more than one quaternion component is supposed to convey breathing information, the possibility to maximize this information fusing the 4 components of the quaternion by means of PCA was investigated. PCA is a mathematical procedure that transforms an original set of correlated variables into a (smaller) number of uncorrelated variables by determining a set of orthogonal vectors called principal components, which are defined by a linear combination of the original variables [30,31]. To do this, the directions in the data with the most variation, i.e., the eigenvectors corresponding to the largest eigenvalues of the covariance matrix, are computed and the data are projected onto these directions. To compute the eigenvectors, data were arranged into a two-dimensional matrix **X**(*m* × *n*), where *m* was the number of observations of the time series and *n* the number of variables (quaternion components). Then, the univariate means were subtracted from the *n* columns, to center the data. Singular Value Decomposition (SVD) was used to compute the eigenvectors (**V** = [v_1_, v_2_, v_3_, v_4_]) and corresponding eigenvalues (λ_1_, λ_2_, λ_3_, λ_4_). Original data were finally projected in the new coordinate system (**Y** = **XV**) and the first principal component, accounting for the largest possible variance, was selected and passed to other blocks [30,31].

**Spectrum Analysis** block include a set of steps needed to optimize the subsequent processing phase. The two signals representing chest-wall (abdominal and thoracic) respiratory-related orientation obtained downstream of the dimension-reduction block underwent the following steps (Figure 1):

(i). A low-frequency threshold (f_LOW_) was determined based on a first estimate of the breathing frequency (f_B_). The rough estimate of f_B_ was done by identifying maxima points of the signal and computing the f_B_, breath by breath, as reciprocal of the temporal distance between consecutive maxima points. Then, the mean (f_B_Rough_) and the standard deviation (f_B_Rough_SD_) of the f_B_ over the entire trial were computed. To facilitate maxima points identification, signals were at first band-bass filtered using a first-order infinite impulse response (IIR) Butterworth filter [0.05 Hz–2 Hz] and smoothed with a third-order Savitzky–Golay [32] finite impulse response (FIR) filter (fixed window length = 31 samples). Low thresholds f_LOW_Ab and f_LOW_Th were determined for the abdominal and thoracic signals respectively as difference f_B_Rough_ − f_B_Rough_SD_. Then the minimum value between f_LOW_Ab and f_LOW_Th was chosen as final low-frequency threshold, named f_LOW_, and it was used in the next step.
(ii). PSD estimate (Welch’s method, Hamming window size: 300 samples, overlapping: 50 samples) was computed and the spectrum frequency corresponding to the breathing rate was identified, both for the thorax (f_peak_T_) and the abdomen (f_peak_A_), by looking for the local peak of the PSD within the window [f_LOW_ ÷ 2 Hz]. The use of a low threshold, based on a rough estimate of the breathing frequency, supports the selection of the PSD peak linked to breathing rate and avoid selecting wrong peaks, often related to low-frequency oscillation artifacts.
(iii). The breathing frequency derived by the spectrum was used to set an adaptive band-pass filter, as proposed in a previous study [7], centered on f_peak_ frequency. For the abdomen, upper (f_U_) and lower (f_L_) cut-off frequency points for the band-pass filter were defined, by applying Equations (4) and (5) respectively [7]:

f_U_A_ = f_peak_A_ + 0.04, (4)

f_L_A_ = max (0.05, (f_peak_A_ − 0.04)), (5)

For the thorax, Equations (6) and (7) were applied:

f_U_T_ = f_peak_T_ + 0.04, (6)

f_L_T_ = max (0.05, (f_peak_T_ − 0.04)), (7)

Moreover, based on f_peak_, a set of parameters was selected to optimize subsequent smoothing and minima/maxima detection phases of the processing block.

**Processing** block includes all the steps needed to extract breathing frequency and temporal parameters from the signals obtained downstream of the dimension-reduction block. Chest-wall respiratory-related orientation change signals (abdominal and thoracic) underwent the following steps:

(i). Adaptive band-pass filter. The signals were band-pass filtered (first-order IIR Butterworth filter), with f_U_ and f_L_ cut-off frequency points determined within the spectrum analysis block.
(ii). Smoothing. Filtered signals were furtherly smoothed (third-order Savitzky–Golay FIR filter) to simplify subsequent identification of maxima and minima points. The level of smoothing (window length) was automatically selected based on f_peak_, i.e., increasing window length for decreasing f_peak_. Relation between optimal window length values and f_peak_ values has been determined empirically.
(iii). Minima and maxima points detection. A set of optimized parameters (i.e., minimum peak distance (MPD) and minimum prominence threshold (MPT)) was automatically selected based on f_peak_ to optimize recognition of minima and maxima points of the smoothed signals. Optimal MPD and MPT values depending on f_peak_ were experimentally determined.
(iv). Breathing frequency extraction. Breath by breath, inspiratory time (T_I_) was computed as the temporal distance between a minimum point (m_i_) and the consecutive maximum point (M_i_); Expiratory time (T_E_) was computed as the temporal distance between the maximum point (M_i_) and the consecutive minimum point (m_i_ + 1); total time (T_TOT_) was computed as $T_{\mathrm{TOT}}=T_{I}+T_{E}$ [s], duty cycle (DC) was computed as $\frac{T_{I}}{T_{\mathrm{TOT}}}\times 100$ [%] and breathing frequency was computed as $\frac{60}{\left(T_{\mathrm{TOT}}\right)}$ [breaths/minute]. A mean value for each of the above-mentioned parameter was computed for each trial (~3 min).

### 2.4. Experimental Setup

To evaluate the capability of the device and of the proposed methods to correctly estimate breathing frequency (and temporal parameters) 8 healthy volunteers (4 males, 4 females) were enrolled. All subjects gave their informed consent for inclusion before they participated in the study. The study was conducted in accordance with the Declaration of Helsinki, and the protocol (Project identification code n° 534) was approved by the Ethics Committee of Scientific Institute IRCCS Medea (date of approval: 25 January 2018). Chest-wall movements during breathing, in seated and supine position, were measured using the proposed device and optoelectronic plethysmography (OEP) simultaneously. OEP [21] is a technique based on a similar functioning principle of the proposed device; in fact, it allows assessment of ventilatory and breathing pattern by measuring chest-wall movements related to breathing, by using motion capture principles. The system is composed of eight infrared video cameras working at a sampling rate of 60 Hz. It can compute the 3D coordinates of retro-reflective markers positioned on the chest wall in specific anatomic points. From the three-dimensional coordinates of the markers, it is possible to obtain the volume enclosed by the chest-wall surface, by applying the Gauss’s theorem. The chest wall is modelled by a bicompartmental model, composed of rib cage and abdomen, and thus it is possible to investigate the contribution of both the compartments. This is an advantage for the validation of the proposed device, in fact, using OEP as reference method it is possible to compare the data recorded with the thoracic and abdominal units of the device with those obtained by using OEP for the thoracic and abdominal compartments, respectively. OEP has been widely validated against spirometer, in healthy subjects, in different conditions and positions, also during submaximal and maximal exercise on cycle ergometer, obtaining discrepancies in tidal volume measurements always <5% [22,23,24,33,34].

**Figure 3:**
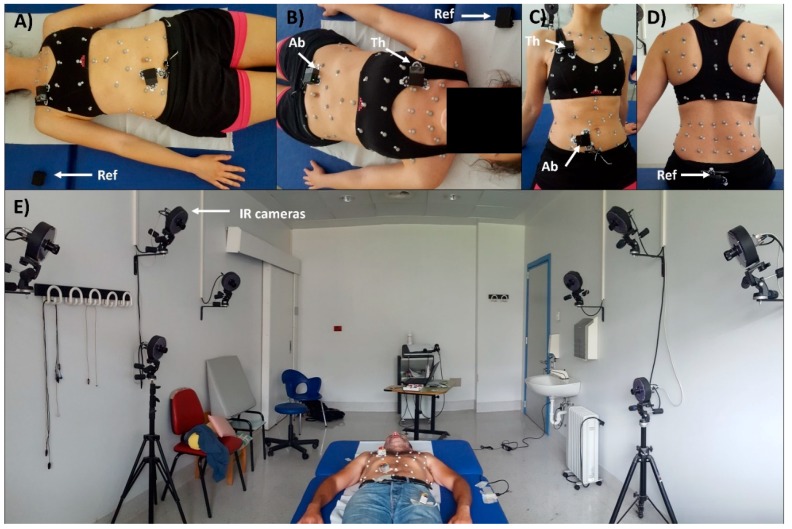
Experimental setup. Retroreflective-marker configuration for optoelectronic plethysmography (OEP) and IMU-unit (Ab: Abdomen, Th: Thorax, Ref: Reference) placement in supine (A and B panels) and seated (C and D panels) positions. Panel E shows the experimental setup and the OEP Lab; Infrared cameras of the motion capture system are also noticeable.

The subjects were prepared, placing the reflective markers according to the 89-marker protocol (previously described in [24,35]) used for seated position and the 52-marker protocol (previously described in [36,37]) used for supine position or, more generally, when a back support is present. Then peripheral IMU-units were placed on the thorax and on the abdomen, while reference IMU-unit was placed on the coccyx in seated position, and on the bed in supine position (Figure 3).

Subjects were then asked to seat or lie on a bed and were invited to perform a slow vital capacity maneuver (SVC) and then to start breathing with the following patterns: (I) quiet breathing (QB), (II) increasing f_B_ but same tidal volume of QB (↑f_B_, V_T_=), (III) increasing f_B_ and reducing tidal volume (↑f_B_, V_T_↓), (IV) decreasing f_B_ with the same tidal volume of QB (↓f_B_, V_T_=), (V) decreasing f_B_ increasing tidal volume of QB (↓f_B_, V_T_↑). QB trial was repeated two times, thus, each subject performed 6 trials of the duration of 3 min each. The SVC maneuver was used to align OEP signal and device signals during data analysis, since it is generally recognizable with respect to QB. In fact, SVC requires a maximal inspiration followed by a complete expiration without forced or rapid effort.

The subjects were asked to maintain the same breathing pattern (namely, QB, ↑f_B_, V_T_=, ↑f_B_, V_T_↓, ↓f_B_, V_T_=, ↓f_B_, V_T_↑) until the end of the trial; in case of fatigue they were asked to perform a second SVC before returning to QB. This procedure was repeated in seated position and in supine position.

### 2.5. Statistical Analysis

For each trial, mean values of f_B_, T_I_, T_E_ and DC were extracted from the best quaternion components identified online by using “Area” and “Peak” methods, and from the signal obtained with the PCA-based fusion method, both for the thoracic and abdominal tracings. Moreover, to evaluate the performance of the selection methods (“Area” and “Peak”) and their ability to select the best component, the same parameters were obtained for all the quaternion components (q_0_, q_1_, q_2_, q_3_) and compared with those obtained by OEP, on the abdominal and thoracic compartment, respectively. The “Ideal” quaternion component was identified a posteriori, trial by trial, as the one providing the minimum estimation error of the breathing frequency. Obviously, the “Ideal” component cannot be identified during online analysis, or when a reference method is not present. Thus, for each trial, 5 sets of parameters were available:

- f_B_OEP_, T_I_OEP_, T_E_OEP_ and DC__OEP_
- f_B_Peak_, T_I_Peak_, T_E_Peak_ and DC__Peak_
- f_B_Area_, T_I_Area_, T_E_Area_ and DC__Area_
- f_B_PCA_, T_I_PCA_, T_E_PCA_ and DC__PCA_
- f_B___Ideal_, T_I___Ideal_, T_E___Ideal_ and DC____Ideal_

Among the entire set of trials, those with f_B_OEP_ < 6 breaths/minute or f_B_OEP_ > 60 breaths/minute were discarded. Then, the absolute (Equation (8)) and relative (Equation (9)) errors of estimation in static conditions (supine and seated position) were computed for each parameter:

(8) Absolute Error (E)=|Device−OEP|,

(9) $$Relative\;Error\;\left(E\%\right)=\frac{\left|Device-OEP\right|}{OEP}\times 100$$

For all the dimension-reduction methods (“Area”, “Peak”, “PCA”), mean and standard deviation (SD) were computed for E and E% considering all the subjects and all the trials, for the supine and seated position and compared with those obtained considering the “Ideal” component. The error obtained with the “Ideal” component, identified a posteriori, is thus the minimum error obtainable using a single quaternion component, and represents the performance that the other methods (“Area”, “Peak”, and PCA-fusion) should achieve or beat. For E% obtained in f_B_ and DC estimation, non-parametric alternative to the one-way Analysis of variance (ANOVA) with repeated measures (Friedman test) was performed to assess if significant differences between methods (“Area”, “Peak”, “PCA”) and “Ideal” component occurred, “Ideal”); post-hoc analysis was done performing Wilcoxon signed-rank tests on the different combinations of related methods, applying the correction for multiple comparisons using false discovery rate (FDR) method [38,39].

For f_B_, T_I_, T_E_, linear regression analysis and correlation analysis (Pearson’s product-moment correlation r_P_, or Spearman’s rank-order correlation r_S_, if data were not normally distributed) were performed between measurements obtained with the device and measurements obtained with the OEP, for the supine and seated position, respectively.

To assess the agreement between measurements obtained with the device and with the OEP, Bland–Altman analysis was performed plotting the difference of the two paired measurements (device–OEP) against the mean of the two measurements [40,41,42]. Mean of the differences (d) and limits of agreement (LOA: from d − (1.9 × SD) to d + (1.9 × SD)) were calculated. The presence of heteroscedasticity was always examined to assess the presence of proportional biases and/or the correlation between differences and mean values. As proposed by Brehm et al. [43], to determine if data were heteroscedastic a visual inspection of Bland–Altman plots was performed at first. If the errors (*y*-axes: absolute differences) increased with increasing measured values (*x*-axes: mean), the data were suspected of being heteroscedastic. Then Kendall’s tau (τ) correlation between the absolute differences and the corresponding means was computed to assess the degree of heteroscedasticity. Data were denoted heteroscedastic when a positive, significant correlation (τ > 0.1 and p-value < 0.05) was found, for other cases data were considered homoscedastic [43].

When heteroscedasticity was present the “classical” 95% confidence and tolerance limits cannot be constructed, thus the approach based on the construction of V-shaped limits was applied: the regression line (ordinary least squares (OLS) best fit) was constructed for differences on mean values and the V-shaped confidence limits (upper confidence limit: UCL, lower confidence limit: LCL) were constructed modelling the variability in the SD of the differences directly as a function of the level of the measurement, using a method based on absolute residuals from a fitted regression line [44,45].

## 3. Results

### 3.1. Breathing Patterns

Table 1 presents the mean and SD of breathing rate for each breathing pattern (QB_1_ e QB_2_, ↑f_B_, V_T_=, ↑f_B_, V_T_↓, ↓f_B_, VT=, ↓f_B_, V_T_↑) estimated with OEP and device, using “PCA”, “Area”, and “Peak” methods and the “Ideal” component, for all subjects, in supine and seated position. Sample size (n) of each condition is reported in Table 1 for the breathing pattern ↑f_B_, V_T_↓, just one thoracic tracing was available for seated position (n = 1). It can be noticed that each subject demonstrated a different breathing frequency for each breathing pattern and SD in the forced breathing patterns is higher than those obtained for QB, meaning that subjects interpreted the required speed differently.

### 3.2. Accuracy Errors

**Figure 4:**
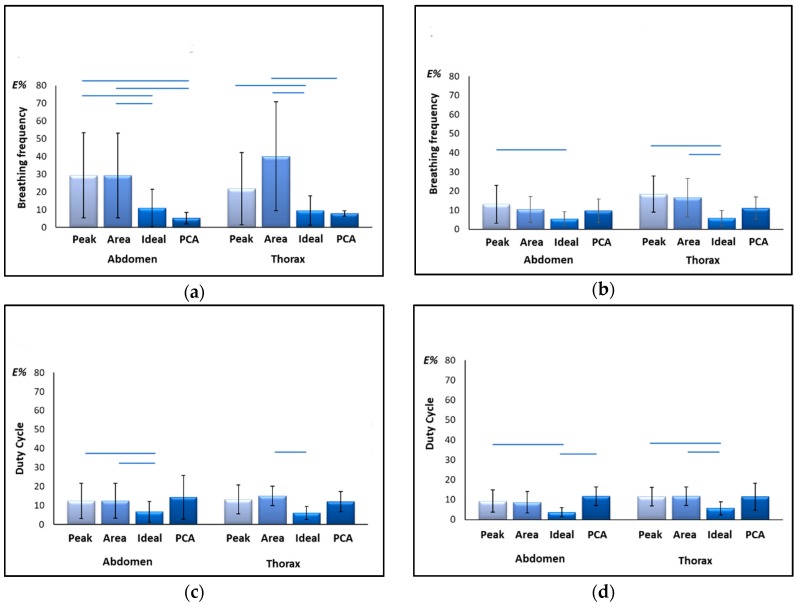
Relative errors (E%) of estimation of breathing frequency (**a**,**b**) and Duty Cycle (**c**,**d**) in supine (**a**,**c**) and seated (**b**,**d**) positions, computed for each method (Peak, Area and PCA) and for the “Ideal” component with respect to the reference (OEP). Errors are computed both for the Thoracic and abdominal compartments. Horizontal blue lines indicate statistical significance of difference (post-hoc analysis, Wilcoxon test FDR corrected).

Relative errors of estimation in supine and seated position computed for best component-selection methods (“Peak”, “Area”), for PCA-fusion method and for the “Ideal” quaternion component for f_B_ and DC are presented in Figure 4. For what concerns f_B_ estimation in supine position, relative errors obtained using PCA were similar or even better than those provided by the “Ideal” component; on the contrary, both component-selection methods, namely “Area” and “Peak”, provided errors higher than 10%, both for the abdominal and the thoracic compartments. Errors obtained with PCA resulted significantly lower than those obtained with the “Area” method, both for the abdominal (Wilcoxon post-hoc test FDR-adjusted, p = 0.038) and thoracic compartment (Wilcoxon post-hoc test FDR-adjusted, p = 0.015); also, PCA was significantly better than “Peak” method considering abdominal compartment (Wilcoxon post-hoc test FDR-adjusted, p = 0.038). Errors obtained with “Ideal” component resulted significantly lower than those obtained with the component-selection methods both for the abdominal (Wilcoxon post-hoc test FDR-adjusted, Ideal vs. Area p = 0.038, Ideal vs. Peak p = 0.038) and thoracic (Wilcoxon post-hoc test FDR-adjusted, Ideal vs. Area p = 0.000, Ideal vs. Peak p = 0.020) compartments.

In seated position, f_B_ estimation errors obtained with component-selection methods were lower on average than those obtained in supine position, while PCA performances declined. This led to a sort of equalization effect, confirmed also by the statistical analysis: significant differences remained only for comparisons “Ideal” vs. “Area” method (Wilcoxon post-hoc test FDR-adjusted, AB: p = 0.102, TH: p = 0.015) and “Ideal” vs. “Peak” method (Wilcoxon post-hoc test FDR-adjusted, AB: p = 0.006, TH: p = 0.015).

Regarding duty cycle, relative errors of estimation obtained with the different methods are comparable, with exception of those provided by “Ideal” component that are on average lower, both in supine (Wilcoxon post-hoc test FDR-adjusted, AB: Ideal vs. Peak p = 0.042, Ideal vs. Area p = 0.042; TH: Ideal vs. Area p = 0.006) and seated position (Wilcoxon post-hoc test FDR-adjusted, AB: Ideal vs. Peak p = 0.015, Ideal vs. PCA p = 0.006; TH: Ideal vs. Area p = 0.015, Ideal vs. Peak p = 0.015, Ideal vs. PCA p = 0.05).

Absolute estimation errors of f_B_, T_I_ and T_E_ obtained with the device using different methods (Area, Peak, Ideal, PCA) relative to OEP are reported in Table 2.

### 3.3. Linear Regression and Correlation Analysis

**Figure 5:**
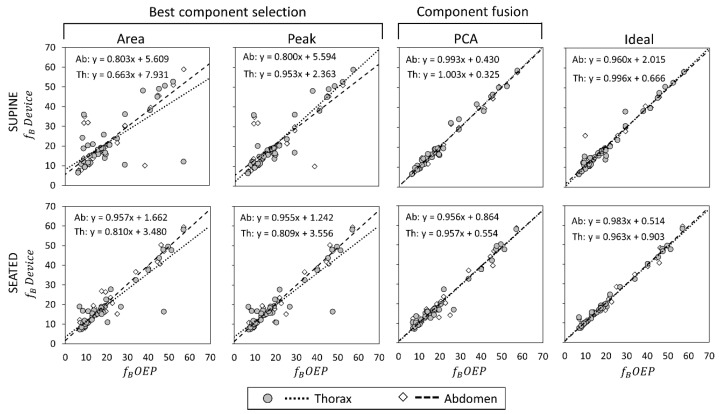
Comparisons of breathing frequency (f_B_ expressed as breaths/minuteute) measurements by using the proposed device and by using Optoelectronic plethysmography (OEP) presented as regression analysis, in supine (top panels) and seated (bottom panels) positions. For what concerns f_B_ measurements obtained with the IMU-device, three dimension-reduction methods were considered: Area, Peak and PCA-fusion. The performance obtained by using these three methods is benchmarked against that obtained with the Ideal quaternion component determined a posteriori based on the minimum estimation error. The regression line between measurements done by OEP and the proposed device is plotted, and the relative equation presented, both for the thorax and the abdomen.

**Figure 6:**
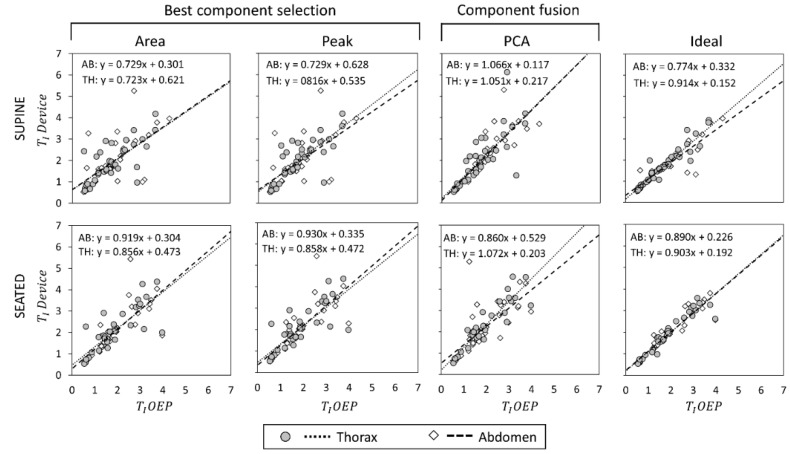
Comparisons of inspiratory time (T_I_ expressed as seconds) measurements by using the proposed device and by using Optoelectronic plethysmography (OEP) presented as regression analysis, in supine (top panels) and seated (bottom panels) positions. For what concerns T_I_ measurements obtained with the IMU-device, three dimension-reduction methods were considered: Area, Peak and PCA-fusion. The performance obtained by using these three methods is benchmarked against that obtained with the Ideal quaternion component determined a posteriori based on the minimum estimation error. The regression line between measurements done by OEP and the proposed device is plotted, and the relative equation presented, both for the thorax and the abdomen.

**Figure 7:**
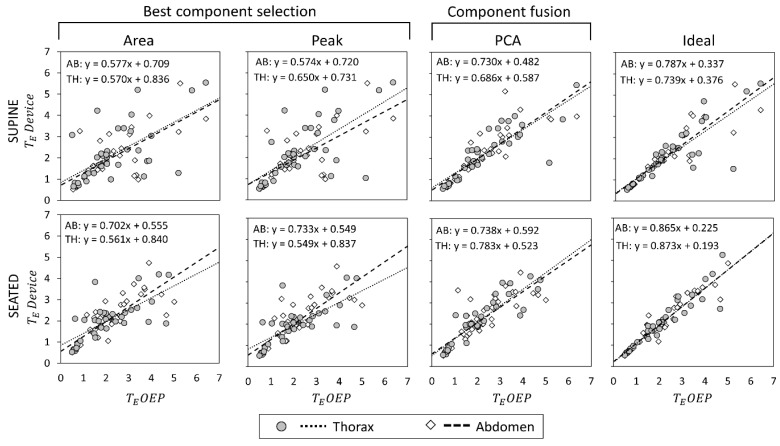
Comparisons of expiratory time (T_E_, expressed as seconds) measurements by using the proposed device and by using Optoelectronic plethysmography (OEP) presented as regression analysis, in supine (top panels) and seated (bottom panels) positions. Regarding T_E_ measurements obtained with the IMU-device, three dimension-reduction methods were considered. Area, Peak and PCA-fusion. The performance obtained by using these three methods is benchmarked against that obtained with the Ideal quaternion component determined a posteriori based on the minimum estimation error. The regression line between measurements done by OEP and the proposed device is plotted, and the relative equation presented, both for the thorax and the abdomen.

Scatter plots showing the relationship between measurements obtained with the OEP and with the device, using “Area”, “Peak”, “Ideal” components, and PCA-fusion respectively are presented for f_B_ (Figure 5), T_I_ (Figure 6) and T_E_ (Figure 7). For each scatter plot the regression line is computed, both for thorax and abdomen, and the relative equations are reported.

Correlation coefficients for the comparisons Device vs. OEP are reported in Table 3. Regarding the main parameter, f_B_, results obtained with correlation analysis confirmed what emerged from estimation error analysis: in supine position, PCA exhibited the best performances in terms of correlation with OEP measurements both in terms of regression line and correlation coefficient. In seated position, “Ideal” component was the one with the highest correlation with OEP measurements, followed by PCA.

With reference to T_I_ estimation in supine position, “Ideal” component provided the best performances, followed by PCA method; “Peak” and “Area” methods provided comparable, poor performances. In seated position, measurements of T_I_ provided by component-selection methods were on average more correlated with OEP measurements than measurements obtained using PCA-fusion method. The “Ideal” component presented the best results, followed by “Area” and “Peak” methods; PCA provided the worst performance considering the abdominal compartment, while correlation between measurements obtained with the thoracic unit and OEP measurements was good.

Estimation of T_E_ was on average more problematic. In terms of regression lines in fact, slope values were far from the unity for all the considered methods, highlighting a proportional error leading to an overestimation for low values of expiratory time and an underestimation at high expiratory times, as shown in Figure 7. For what concerns supine position, correlation coefficients were good both for “Ideal” component and PCA-fusion method; on the contrary, correlation coefficients were low both for “Area” and “Peak” methods. Also, in seated position correlation coefficients provided by “Ideal” and PCA-fusion method were higher than those provided by “Area” and “Peak” methods, especially with respect to the thoracic compartment.

### 3.4. Bland–Altman Analysis

**Figure 8:**
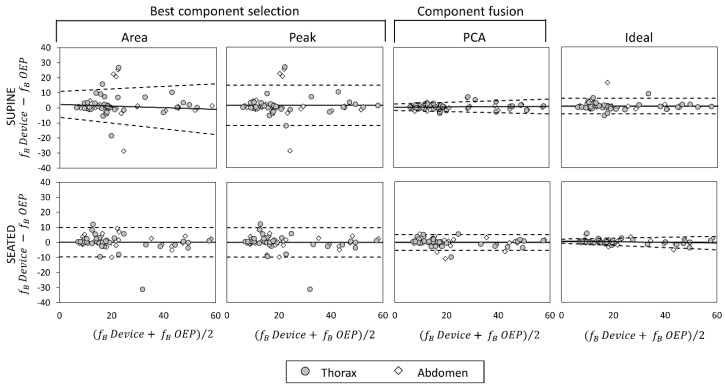
Agreement analysis between OEP and the IMU-based device for breathing frequency (f_B_, expressed as breaths/minuteute) measurements, in supine (top panels) and seated (bottom panels) position. In each Bland–Altman plot the differences between measurements of f_B_ obtained by using the IMU-based device and by using OEP are plotted against the mean of the two measurements. For homoscedastic data, the mean of the differences (bias: —) and limits of agreement (black dotted line) from mean − 1.96 s to mean + 1.96 s are represented by lines parallel to the X axis. For heteroscedastic data, the proportional bias (—) is represented by the ordinary least squares (OLS) line of best fit for the difference on mean values; V-shaped upper and lower 95% confidence limits (- - -) are calculated according to Bland [44].

**Figure 9:**
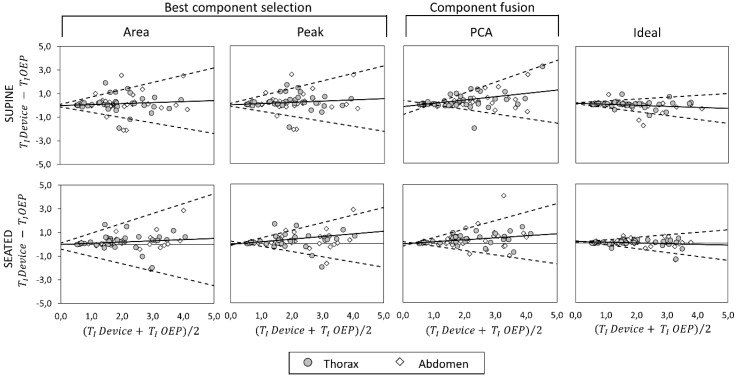
Agreement analysis between OEP and the IMU-based device for inspiratory time (T_I_, [s]) measurements, in supine (top panels) and seated (bottom panels) position. In each Bland–Altman plot the differences between measurements of T_I_ obtained by using the IMU-based device and by using OEP are plotted against the mean of the two measurements. For homoscedastic data, the mean of the differences (bias: —) and limits of agreement (- - -) from mean − 1.96 s to mean + 1.96 s are represented by lines parallel to the X axis. For heteroscedastic data, the proportional bias (—) is represented by the OLS line of best fit for differences on mean values; V-shaped upper and lower 95% confidence limits (- - -) are calculated according to Bland [44].

**Figure 10:**
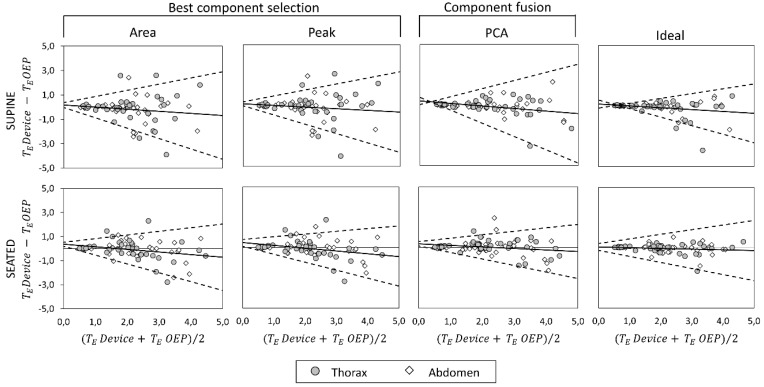
Agreement analysis between OEP and the IMU-based device for expiratory time (T_E_, [s]) measurements, in supine (top panels) and seated (bottom panels) position. In each Bland–Altman plot the differences between measurements of T_E_ obtained by using the IMU-based device and by using OEP are plotted against the mean of the two measurements. For homoscedastic data, the mean of the differences (bias: —) and limits of agreement (- - -) from mean − 1.96 s to mean + 1.96 s are represented by lines parallel to the X axis. For heteroscedastic data, the proportional bias (—) is represented by the OLS line of best fit for differences on mean values; V-shaped upper and lower 95% confidence limits (- - -) are calculated according to Bland [44].

Bland–Altman plots representing the agreement between measurements obtained with the OEP and with the device, using “Area”, “Peak”, “Ideal” components, and PCA-fusion respectively are presented for f_B_ (Figure 8), T_I_ (Figure 9) and T_E_ (Figure 10). In Bland–Altman plots, the difference of the two paired measurements (device–OEP) is plotted against the mean of the two measurements (device+OEP)⁄2. Results of agreement analysis, including evaluation of heteroscedasticity (Kendall’s τ correlation and relative p-value) are reported in Table 4. As shown there, for homoscedastic data, the mean of the differences representing the fixed bias, and LOAs were computed. On the other hand, for heteroscedastic data, OLS line of best fit representing the proportional bias and upper and lower 95% V-shape confidence limits (UCL and LCL) are reported.

With respect to the main parameter (f_B_), agreement between OEP and the device is very strong when the “Ideal” component or the PCA-fusion are used, both in supine and seated position. In relation to time estimation, the agreement decreases for all the methods considered. In particular, for what concerns inspiratory times, a significant relationship between errors and mean value emerged, with a general increase of the difference (device–OEP) at higher time values (overestimation of the device), both in supine and seated position. Also, for expiratory times absolute errors increased with increasing time values, but in this case the device underestimated at high time values (negative slope of the OLS).

### 3.5. Quaternion Component Selection 

**Figure 11:**
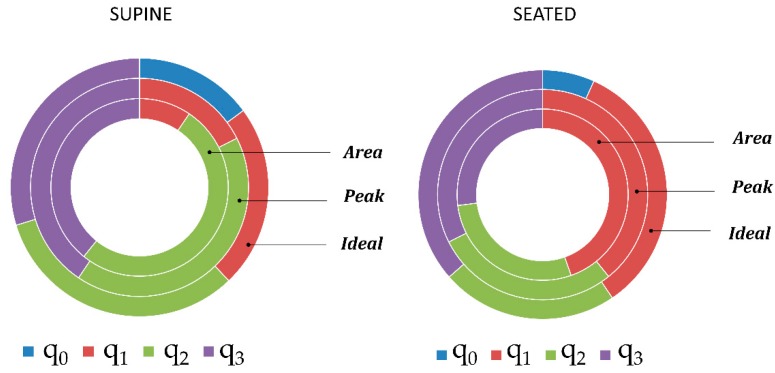
Relative frequencies of quaternion component (q_0_, q_1_, q_2_, q_3_) selection using Area and Peak methods and of quaternion component selection as Ideal component, in supine and seated position. Each portion of the rings represents the ratio between the number of times that each quaternion component has been selected (by Area and Peak methods or as Ideal component respectively) and the total number of trials (n = 74).

Considering the quaternion components selected by the “Area” and “Peak” methods as best component or identified as “Ideal” component, a clear rule did not emerge. In fact, there was not a quaternion component that was selected as best component with a considerable frequency. Relative frequencies of quaternion component selection with the different methods are presented in Figure 11. It is interesting to notice that quaternion component q_0_ was never selected by “Area” and “Peak” methods, while the “Ideal” component was q_0_ in 14.86 % of cases (n = 74) in supine position and 6.76% of cases (n = 74) in seated position. In seated position, the component q_1_ was selected more frequently as best component both by using “Area” (44.59) and “Peak” (39.19%) methods. In contrast, in supine position, the components q_2_ (“Area” 51.35%, “Peak”: 41.89) and q_3_ (“Area”: 39.19% and “Peak”: 40.54%) were selected more frequently.

Excluding q_0_ component, that was clearly less selected, the other quaternion components were almost equally selected as “Ideal” component considering all the trials, both in supine position (q_0_: 14.86%, q_1_: 22.97%, q_2_: 32.43%, q_3_: 29.73%), and seated position (q_0_: 6.76%, q_1_: 33.78, q_2_: 22.97%, q_3_: 36.49%).

In regard to the ability of the two component-selection methods (“Area” and ”Peak”) to identify the “Ideal” component, i.e., the component providing the minimum f_B_ estimation error, in supine position, the “Area” method was able to identify the “Ideal” component in 45.94% of the cases (relative frequency for the event “the component selected by “Area” method and the “Ideal” component corresponded”), the “Peak” method identified the “Ideal” component in 52.70% of the cases, while for the 45.94% of the cases neither the “Area” method nor the “Peak” method were able to identify the “Ideal” component. In 44.59% of cases, “Area” method and “Peak” method selected the same quaternion component, that was also identified as “Ideal” component.

## 4. Discussion

In this study, we presented an automatic, position-independent processing algorithm to derive breathing signal, and subsequently breathing temporal parameters, from chest-wall orientation changes acquired using an IMU-based device previously developed by our group [19], composed of three sensor units. Even if the modular configuration of the device was designed to reduce non-breathing movements, the aim of this work was neither to demonstrate the effectiveness of this approach nor to support the presence of the reference unit. On the contrary the focus is on the analysis algorithm, which uses quaternion form to represent orientation, avoiding singularity problem that affects Euler representation; thus, thoracic and abdominal orientation change signals are 4-dimensional entities. The proposed algorithm includes therefore a dimension-reduction block to obtain a 1-dimension signal representing chest-wall orientation changes due to breathing activity.

Another aim of this study was, therefore, to compare three different dimension-reduction methods. The first two methods (“Peak” and “Area”) are based on the selection of the quaternion component with the highest breathing information. The third method is based on the fusion of the 4 components of the quaternion using PCA.

The PCA-fusion method performed better than the best component-selection methods (“Peak” and “Area”) as regards breathing frequency estimation, both in supine and seated position. In supine position, it provided better results than “Ideal” component, while in seated position it provided closer performances to those obtained by using the “Ideal” component with respect to “Area” and “Peak” methods.

About estimation of other temporal parameters (T_I_, T_E_ and duty cycle), “Ideal” component provided the best results, while PCA-fusion method gave results comparable to the best component-selection methods. Thus, a quaternion component providing the best performance exists, the problem lies in its a priori identification. In fact, both “Area” and “Peak” methods failed to identify it on the basis of spectral analysis (in 45.94% of the cases neither the “Area” method nor the “Peak” method were able to identify the “Ideal” component), and no quaternion component emerged as the most selected as “Ideal” component (supine q_0_:14.86%, q_1_: 22.97%, q_2_: 32.43%, q_3_: 29.73%; seated q_0_: 6.76%, q_1_: 33.78, q_2_: 22.97%, q_3_: 36.49%).

Geometrical or morphological considerations to determine which quaternion component is more involved in breathing movement are problematic when considering quaternions, and are position- and IMU-placement dependent, thus not suitable in dynamic conditions. On the contrary, PCA-fusion method represents an interesting solution to this problem because it fuses the information of the four quaternion components regardless the position of the subject or the IMUs placement, avoiding the necessity to select a best component/axes, as reported in previous studies [7,11,13].

In this study, we found that PCA-fusion method provided the best f_B_ estimation performance in terms of mean absolute errors (<2 breaths/minute), correlation (r > 0.963) and agreement (see Table 4) with the reference method. Comparing our results in terms of accuracy errors with those obtained by previous studies is difficult because in most cases only relative errors were reported, but these errors depend on the breathing frequency adopted. Liu et al. [14] reported a mean absolute error of 15.45 breaths/minute (thus about 7 times higher than the error obtained in this study) during quiet sitting. Bates et al. [13] obtained an RMS error of 0.38 breaths/minute and a peak error of 3 breaths/minute in a postoperative patient during sleep. Considering comparable conditions (abdominal compartment in supine position) we obtained an RMS error of 1.51 breaths/minute, using PCA-fusion method, but in our study different, forced, breathing patterns were included, leading to higher mean error.

Regarding correlation between f_B_ measurements obtained with the proposed method and OEP, our results are comparable to those obtained by Bates et al. [13] that reported a correlation coefficient equal to 0.985 between measurements of f_B_ obtained with the accelerometer and nasal cannula. Mann et al. [11] obtained a correlation coefficient of 0.97 between measurements of f_B_ obtained with a tri-axial accelerometer and with a system based on oxygen consumption measurement (Oxycon Mobile). In both cases [11,13], breath-by-breath analysis was not possible. Liu et al. [14] reported correlation coefficients lower than 0.6 between f_B_ computed with a 3-axis accelerometer and with the reference (Airflow CO_2_ analysis).

There are few studies in the literature that performs Bland–Altman analysis to assess the agreement between breathing frequency measurements obtained by using inertial sensors and other validated methods. In the study from Morillo et al. [8] agreement analysis using Bland Altman plots was done against PSG thermistor. The authors reported a mean difference (fixed bias) of 0.02 (SD = 1.09) breaths/minute and LOAs from −3.05 to +2.11 breaths/minute in the range ~12 ÷ 35 breaths/minute. In that case, the use of a single-axis accelerometer, prevent the use of that method during postural changes. Dehkrodi et al. [9] used a tri-axial accelerometer placed on the suprasternal notch extending the validation presented in [8] to different sleeping positions and breathing conditions (Deep: 13.5 ± 4.3, Normal: 16.5 ± 5.2 and Shallow: 39.7 ± 30.3 breath/minute). They reported a mean difference (fixed bias) of 0.042 breaths/minute and LOAs from –0.65 to 0.74 breaths/minute. Lapi et al. [17] performed agreement analysis with Bland Altman plots for measurements of breathing frequency (range 12 ÷ 26 breaths/minute) obtained with the accelerometer and with the standard method (counting breaths by visual inspection), in supine position. They reported a mean difference (fixed bias) of 0.33 breaths/minute and LOA from −1.92 to 2.60 with 3.2% of data outside that range. For all the above-mentioned studies heteroscedasticity of data was not considered or reported making it difficult compare them directly with our results. In fact, taking into account heteroscedasticity of data, for f_B_ in supine position, we built Bland–Altman plot with proportional bias (OLS: y = 0.008x + 0.130, thus going from 0.18 (at x = 6 breaths/minute) to 0.61 (at x = 60 breaths/minute) breaths/minute) and V-shaped limits (LCL: y = −0.038x − 2.039 thus the lower limit goes from −2.26 (at x = 6 breaths/minute) to −4.32 (at x = 60 breaths/minute) breath/minute; UCL: y = 0.054x + 2.299; thus the upper limit goes form 2.62 (at x = 6 breaths/minute) to 5.539 (at x = 60 breaths/minute) breaths/minute) ). Thus, considering comparable breathing frequency ranges our results are closer to those obtained by Morillo et al. [8] and Lapi et al. [17]. On the contrary, Dehkrodi et al. [9] obtained better results; unfortunately, the steps to obtain the acceleration derived respiratory (ADR) signal are not described in detail.

For the best of our knowledge, this is the first study that provides a detailed analysis of respiratory timing measurements obtained by using inertial sensor systems, validating them against an established method.

## 5. Conclusions

PCA-fusion method provided overall best performances with respect to selecting the best quaternion component identified based on spectrum analysis. In supine position results obtained fusing the 4 quaternion components were even better than those obtained with the “Ideal” component, identified a posteriori considering the minimum breathing frequency estimation error. Performance in seated position were worse than those obtained in supine position, probably because subjects were seated without the back support and some oscillations of the trunk were more likely to occur. This could particularly affect PCA-based method where the first principal component selected for further analysis is the one with the largest variance, and thus more subject to larger body motions. This must be taken into account in dynamic conditions; signal baseline removal prior to PCA-fusion should be considered in this case.

The analysis algorithm proposed in this work, applying PCA-fusion as dimension-reduction method, can be used to analyze further data. In fact, an extended validation of the proposed device and method is needed also in dynamic conditions, during daily activities, considering not only healthy subjects but also patients that could particularly take advantage of this system (e.g., COPD, neuromuscular patients, sleep apnea, etc.). This would also allow study of asynchronies of thoraco-abdominal compartments taking advantage of the modular configuration of the device. Another key step will be the adaptation of our method, currently implemented as an offline analysis, to online monitoring, moving the computation process aboard the smartphone. This enhancement could allow immediate computation of an average breathing frequency over a certain period (e.g., 60 s) directly aboard the smartphone, fostering the use of the device in other applications such as sport and fitness, exercise testing, breathing training to use different respiratory muscles, rehabilitation protocols and treatment evaluation where respiratory assessment could be of great interest.

## 6. Patents

The present work is partially described in the International Patent application n° PCT/IB2018/054956, priority date 11 July 2017, title “A wearable device for the continuous monitoring of the respiratory rate”. Inventors: Ambra Cesareo, Andrea Aliverti, Assignee: Politecnico di Milano.

## Figures and Tables

**Table 1:** Breathing frequencies for different breathing patterns.

**Supine**		**QB 1**		**↑f_B_, V_T_=**		**↓f_B_, V_T_=**		**↑f_B_, V_T_↓**		**↓f_B_, V_T_↑**		**QB 2**	
		**AB (n = 8)**	**TH (n = 7)**	**AB (n = 8)**	**TH (n = 8)**	**AB (n = 6)**	**TH (n = 6)**	**AB (n = 3)**	**TH (n = 2)**	**AB (n = 5)**	**TH (n = 6)**	**AB (n = 8)**	**TH (n = 8)**
OEP		17.13 ± 2.23	17.62 ± 2.21	39.49 ± 10.26	39.48 ± 10.25	11.17 ± 2.64	11.14 ± 2.63	48.11 ± 13.29	47.66 ± 13.95	8.38 ± 2.40	8.61 ± 2.24	15.29 ± 4.34	15.24 ± 4.48
Device	Area	17.20 ± 2.15	18.53 ± 3.96	39.19 ± 10.84	38.16 ± 14.67	15.52 ± 8.14	19.29 ± 9.42	34.47 ± 34.58	30.01 ± 25.52	10.11 ± 3.91	10.62 ± 4.44	21.28 ± 8.14	16.55 ± 3.62
	Peak	17.20 ± 2.15	16.63 ±2.29	38.92 ± 11.12	38.96 ± 13.01	15.52 ± 8.14	16.97 ± 9.59	34.47 ± 34.58	53.39 ± 7.55	10.11 ± 3.91	9.32 ± 2.12	21.25 ± 8.15	16.55 ± 3.62
	PCA	17.23 ± 2.09	17.03 ± 1.98	40.06 ± 9.22	40.63 ± 8.45	11.51 ± 2.59	11.56 ± 3.04	48.61 ± 12.64	49.93 ± 11.94	8.61 ± 2.19	8.95 ± 2.06	15.11 ± 4.82	15.23 ± 3.79
	Ideal	17.57 ± 2.52	16.34 ± 2.00	39.81 ± 10.50	40.94 ± 9.97	12.11 ± 1.99	13.60 ± 2.61	49.53 ± 12.31	48.12 ± 13.89	9.34 ± 3.33	8.83 ± 2.06	17.74 ± 4.70	15.37 ± 3.82
**Seated**		**QB 1**		**↑f_B_, V_T_=**		**↓f_B_, V_T_=**		**↑f_B_, V_T_↓**		**↓f_B_, V_T_↑**		**QB 2**	
		**AB (n = 8)**	**TH (n = 8)**	**AB (n = 8)**	**TH (n = 7)**	**AB (n = 8)**	**TH (n = 8)**	**AB (n = 2)**	**TH (n = 1)**	**AB (n = 6)**	**TH (n = 5)**	**AB (n = 7)**	**TH (n = 6)**
OEP		16.99 ± 2.65	16.94 ± 2.77	42.74 ± 10.97	46.49 ± 7.58	14.57 ± 13.40	14.49 ± 13.41	33.20 ± 16.01	22.01	10.04 ± 3.56	10.05 ± 3.81	18.47 ± 4.32	18.47 ± 4.49
Device	Area	17.15 ± 2.86	15.78 ± 3.62	43.06 ± 11.22	40.71 ± 13.59	15.71 ± 13.52	17.06 ± 13.05	32.63 ± 12.87	27.62	10.83 ± 2.93	12.58 ± 4.08	19.80 ± 5.15	17.04 ± 2.09
	Peak	16.48 ± 3.58	16.27 ± 3.76	43.06 ± 11.22	40.71 ± 13.59	16.53 ± 13.50	17.15 ± 12.99	32.63 ± 12.87	27.62	10.83 ± 2.93	12.58 ± 4.08	17.23 ± 2.19	16.54 ± 1.95
	PCA	16.54 ± 3.01	16.58 ± 2.10	42.26 ± 11.38	45.31 ± 8.56	16.07 ± 13.07	14.91 ± 13.39	33.79 ± 14.50	27.27	10.76 ± 2.90	11.35 ± 3.73	16.59 ± 2.17	15.47 ± 1.69
	Ideal	16.68 ± 2.59	16.61 ± 2.34	42.64 ± 11.21	45.42 ± 7.98	15.29 ± 13.33	11.07 ± 2.36	33.38 ± 13.92	24.53	10.40 ± 3.53	10.29 ± 3.66	19.06 ± 4.89	18.73 ± 5.04

Across subject mean ± SD of the breathing frequency (f_B_, [breaths/minute]) measurements with OEP and the device, using best component-selection methods (“Area” and “Peak”), PCA-fusion method and “Ideal” component, for the requested patterns. Data are reported for the supine and seated positions, subdivided in abdominal and thoracic contributions.

**Table 2:** Absolute errors of breathing frequency (*E*_f_B_), Inspiratory time (*E*_T_I_) and expiratory time (*E*_T_E_) obtained for the device with respect to OEP, using best component-selection methods (“Area” and “Peak”), PCA-fusion method and “Ideal” component.

			Area	Peak	PCA	Ideal
*E*_f_B_ [breaths/minute]	supine	AB	3.64 ± 7.46	3.64 ± 7.46	1.00 ± 1.24	1.39 ± 2.76
		TH	5.46 ± 8.89	3.17 ± 4.92	1.55 ± 1.51	1.56 ± 1.96
	seated	AB	2.19 ± 2.49	2.12 ± 2.74	1.71 ± 2.25	1.04 ± 1.24
		TH	3.35 ± 5.68	3.31 ± 5.69	1.79 ± 2.04	0.96 ± 0.22
*E*_T_I_ [s]	supine	AB	0.48 ± 0.73	0.48 ± 0.73	0.33 ± 0.51	0.20 ± 0.38
		TH	0.43 ± 0.52	0.41 ± 0.49	0.47 ± 0.67	0.17 ± 0.25
	seated	AB	0.33 ± 0.58	0.36 ± 0.56	0.46 ± 0.71	0.16 ± 0.27
		TH	0.43 ± 0.48	0.44 ± 0.49	0.42 ± 0.35	0.17 ± 0.25
*E*_T_E_ [s]	supine	AB	0.58 ± 0.82	0.58 ± 0.82	0.43 ± 0.58	0.29 ± 0.52
		TH	0.79 ± 0.94	0.67 ± 0.92	0.46 ± 0.63	0.36 ± 0.71
	seated	AB	0.43 ± 0.56	0.43 ± 0.56	0.43 ± 0.55	0.22 ± 0.31
		TH	0.56 ± 0.66	0.56 ± 0.66	0.39 ± 0.41	0.24 ± 0.36

Data are reported as mean ± SD, in supine and seated position for thoracic (TH) and abdominal (AB) compartments.

**Table 3:** Correlation outcomes across subjects and breathing patterns. Coefficient of correlation (r) between measurements obtained using Device vs. OEP are reported for f_B_. T_I_. and T_E_ using best component-selection methods (“Area” and “Peak”), PCA-fusion method and “Ideal” component, in supine (Thorax: n = 37. Abdomen: n = 37) and seated (Thorax: n = 35. Abdomen: n = 39) position.

		Supine		Seated	
		Thorax	Abdomen	Thorax	Abdomen
**f_B_**	**Area**	0.580 ^$^	0.706 ^$^	0.748 ^$^	0.915 ^$^
	**Peak**	0.833 ^$^	0.706 ^$^	0.759 ^$^	0.861 ^$^
	**PCA**	**0.963** ^$^	**0.985** ^$^	0.953 ^$^	0.924 ^$^
	**Ideal**	0.935 ^$^	0.931 ^$^	**0.974** ^$^	**0.977** ^$^
**T_I_**	**Area**	0.727 ^#^	0.665 ^$^	0.812 ^#^	0.812 ^#^
	**Peak**	0.785 ^#^	0.659 ^$^	0.809 ^#^	0.824 ^#^
	**PCA**	0.783 ^#^	**0.874** ^#^	0.926 ^#^	0.731 ^#^
	**Ideal**	**0.943** ^#^	0.862 ^$^	**0.951** ^#^	**0.948** ^#^
**T_E_**	**Area**	0.600 ^$^	0.713 ^#^	0.682 ^#^	0.818 ^#^
	**Peak**	0.687 ^$^	0.712 ^#^	0.723 ^#^	0.835 ^#^
	**PCA**	**0.891** ^#^	0.864 ^#^	0.888 ^#^	0.824 ^#^
	**Ideal**	0.874 ^#^	**0.966** ^$^	**0.938** ^#^	**0.951** ^#^

Correlations are all significant (p-value < 0.001). ^$^ Spearman correlation coefficient; ^#^ Pearson correlation coefficient; Bold: best correlation result.

**Table 4:** Agreement analysis outcomes across subjects and different breathing patterns. Bland and Altman plot statistics for measurements of f_B_. T_I_. and T_E_ using best component-selection methods (“Area” and “Peak”), PCA-fusion method and “Ideal” component and, in supine (n = 74) and seated (n = 74) position.

		τ	p-Value	Heteroscedastic?	Fixed Bias ^a^/OLS	LOA ^c^/V-Shape Limits ^d^
**f_B_ supine**	**Area**	0.159	0.045	Yes	y = −0.054x + 2.316 ^b^	UCL: y = 0.085x + 10.907 ^d^ LCL: y = −0.192x − 6.275
	**Peak**	0.142	0.074	No	1.380 ^a^	From −11.95 to 14.72 ^c^
	**PCA**	0.211	0.008	Yes	y = 0.008x + 0.130 ^b^	UCL: y = 0.054x + 2.299 ^d^ LCL: y = −0.038x − 2.039
	**Ideal**	0.038	0.631	No	0.884 ^a^	From −4.171 to 5.940 ^c^
**f_B_ seated**	**Area**	0.142	0.074	No	0.084 ^a^	From −9.635 to 9.803 ^c^
	**Peak**	0.132	0.096	No	−0.121 ^a^	From −9.931 to 9.688 ^c^
	**PCA**	0.108	0.174	No	−0.23 ^a^	From −5.474 to 5.010 ^c^
	**Ideal**	0.196	0.014	Yes	y = −0.021x + 0.597 ^b^	UCL: y = 0.028x + 2.057 ^d^ LCL: y = −0.071x − 0.864
**T_I_ supine**	**Area**	0.302	0.000	Yes	y = 0.084x − 0.019 ^b^	UCL: y = 0.618x + 0.095 ^d^ LCL: y = −0.450x − 0.132
	**Peak**	0.334	0.000	Yes	y = 0.104x − 0.021 ^b^	UCL: y = 0.638x + 0.093 ^d^ LCL: y = −0.430x − 0.135
	**PCA**	0.375	0.001	Yes	y = 0.283x − 0.175 ^b^	UCL: y = 0.926x − 0.840 ^d^ LCL: y = −0.390x + 0.354
	**Ideal**	0.292	0.000	Yes	y = −0.090x + 0.121 ^b^	UCL: y = 0.158x + 0.163 ^d^ LCL: y = −0.338x + 0.078
**T_I_ seated**	**Area**	0.430	0.000	Yes	y = 0.1022x − 0.0141 ^b^	UCL: y = 0.834x + 0.112 ^d^ LCL: y = −0.618x − 0.409
	**Peak**	0.422	0.000	Yes	y = 0.220x − 0.075 ^b^	UCL: y = 0.642x − 0.173 ^d^ LCL: y = −0.438x + 0.197
	**PCA**	0.489	0.000	Yes	y = 0.171x − 0.0332 ^b^	UCL: y = 0.7182x − 0.226 ^d^ LCL: y = −0.377x + 0.160
	**Ideal**	0.313	0.000	Yes	y = −0.059x + 0.129 ^b^	UCL: y = 0.211x + 0.069 ^d^ LCL: y = −0.329x + 0.189
**T_E_ supine**	**Area**	0.421	0.000	Yes	y = −0.170x + 0.166 ^b^	UCL: y = 0.508x + 0.358 ^d^ LCL: y = −0.847x − 0.026
	**Peak**	0.405	0.000	Yes	y = −0.138x + 0.144 ^b^	UCL: y = 0.496x + 0.306 ^d^ LCL: y = −0.771x − 0.017
	**PCA**	0.522	0.000	Yes	y = −0.209x + 0.328 ^b^	UCL: y = 0.667x + 0.037 ^d^ LCL: y = −1.084x + 0.620
	**Ideal**	0.484	0.000	Yes	y = −0.153x + 0.148 ^b^	UCL: y = 0.3987x − 0.185 ^d^ LCL: y = −0.705x + 0.481
**T_E_ seated**	**Area**	0.384	0.000	Yes	y = −0.216x + 0.364 ^b^	UCL: y = 0.303x + 0.532 ^d^ LCL: y = −0.735x + 0.197
	**Peak**	0.396	0.000	Yes	y = −0.231x + 0.413 ^b^	UCL: y = 0.226x + 0.666 ^d^ LCL: y = −0.657x + 0.101
	**PCA**	0.422	0.000	Yes	y = −0.127x + 0.320 ^b^	UCL: y = 0.284x + 0.498 ^d^ LCL: y = −0.538x + 0.142
	**Ideal**	0.316	0.000	Yes	y = −0.058x + 0.054 ^b^	UCL: y = 0.383x + 0.337 ^d^ LCL: y = −0.500x − 0.228

τ: Kendall’s τ correlation coefficient and relative p-value (heteroscedasticity test). ^a^ Fixed Bias. obtained as the mean of differences (device – OEP). for homoscedastic data. ^b^ OLS: ordinary least squares line of best fit (proportional bias) for heteroscedastic data. ^c^ LOA: limits of agreement. computed as mean difference ± 1.96SD (for homoscedastic data). ^d^ V-shape limits: UCL and LCL 95% confidence limits. calculated according to Bland [44].
